# Intra-cluster correlation coefficients in adults with diabetes in primary care practices: the Vermont Diabetes Information System field survey

**DOI:** 10.1186/1471-2288-6-20

**Published:** 2006-05-03

**Authors:** Benjamin Littenberg, Charles D MacLean

**Affiliations:** 1 General Internal Medicine, University of Vermont, 371 Pearl Street, Burlington, VT, 05401, USA

## Abstract

**Background:**

Proper estimation of sample size requirements for cluster-based studies requires estimates of the intra-cluster correlation coefficient (ICC) for the variables of interest.

**Methods:**

We calculated the ICC for 112 variables measured as part of the Vermont Diabetes Information System, a cluster-randomized study of adults with diabetes from 73 primary care practices (the clusters) in Vermont and surrounding areas.

**Results:**

ICCs varied widely around a median value of 0.0185 (Inter-quartile range: 0.006, 0.037). Some characteristics (such as the proportion having a recent creatinine measurement) were highly associated with the practice (ICC = 0.288), while others (prevalence of some comorbidities and complications and certain aspects of quality of life) varied much more across patients with only small correlation within practices (ICC<0.001).

**Conclusion:**

The ICC values reported here may be useful in designing future studies that use clustered sampling from primary care practices.

## Background

Multi-level or clustered sampling designs are increasingly deployed in medical and health care surveys. In these designs, clusters are identified (*e.g*. medical practices) and then subjects (*e.g*. patients) are sampled from each cluster. The analysis and sample size estimation for such designs must take the clustering into account or the resultant significance tests (*P *values) and confidence intervals will be in error [1]. Generally, failure to account for clustering leads to nominal confidence intervals that are too narrow and to *P *values that are too small. To the extent that patient characteristics are independent of cluster, the effective sample size will be close to the number of individual subjects studied. If the subject characteristics are highly associated within clusters, the effective sample size approaches the number of clusters. In the extreme case, if all the subjects within a cluster are identical, there is no advantage to measuring more than one subject per cluster.

To estimate statistical power or required sample size in a study based on simple random sampling or allocation, one requires an estimate of the minimal important effect and (for continuous measures) the standard deviation of the outcome in the population studied. For clustered designs, however, one must also inflate the sample size to account for the clustering effect. The design effect, sometimes referred to as the variance inflation factor, is a function of the extent of correlation within clusters, the intraclass (or intra-cluster) correlation coefficient (ICC). Unfortunately, pre-study estimates of ICC are difficult to come by and obtaining them constitutes "the main difficulty in calculating sample size for cluster randomized studies" [2].

Several groups have published estimates of ICCs for various patient characteristics observed in large surveys of patients clustered within primary care or general practices from around the world [3-6]. Here we expand their estimates to include those derived from a survey of adults with diabetes clustered within primary care practices in the northeast United States.

## Methods

This study was part of a larger project, the Vermont Diabetes Information System (VDIS), a cluster-randomized trial of a laboratory-based diabetes decision support system in a region-wide sample of 8808 adults with diabetes from 73 Primary Care practices in Vermont and nearby parts of the United States [7]. Primary care in these predominantly rural practices is provided by General Internists, Family Physicians, Physician Assistants, and Nurse Practitioners who provide the bulk of long-term care for these and other patients. There are few diabetes specialists in the region and most diabetes care is provided in the practices. All 119 eligible primary care practices near the thirteen participating hospitals were invited to participate [7]. The participating practices range in size from one provider (in 41 practices) to two practices with six providers each.

A field survey targeted at a sub-sample of subjects was designed to provide a better understanding of the non-laboratory features of the patients before intervention. Field survey subjects were selected at random from the patients participating in the VDIS and invited by telephone to participate in an in-home interview. Patient names were randomly sorted and patients contacted until a sample of approximately 15% of the patients from each practice agreed to an interview. We attempted to contact 4,209 patients and reached 1,576 (37%). Of these, 1,006 (64%) agreed to be interviewed.

Subjects who agreed were mailed a questionnaire and were scheduled for an interview by a trained field interviewer. During the visit, the interviewer reviewed any missing or ambiguous questionnaire items. If necessary, the interviewer read the questions aloud for subjects and recorded their responses for them. Then the interviewer measured the subject as described below and administered a few more instruments that were not included in the questionnaire. The interviews took place during the baseline phase of the study before any interventions were in place. All subjects provided written informed consent. The protocol was approved by the institutional review board of the University of Vermont.

### Demographic, social and economic characteristics

Income was recorded in seven ordered self-reported categories from less than US$15,000 per year to US$100,000 per year or more. Education was also recorded as the highest level completed in seven categories from "Less than 9th Grade" to "Graduate or Professional Degree." We collapsed self-reported race and ethnicity into two categories: Non-Hispanic white and all others. Marital status was collapsed into two categories: Married or living as married *vs*. all others (single, widowed, divorced or separated). We recorded the presence or absence of four types of health insurance: private (commercial indemnity or health maintenance organization benefits often supplied by an employer), Medicare (government health coverage for the elderly and disabled), Medicaid (government health coverage for low income patients), and military (including active duty or veteran's benefits). Subjects may have more than one insurance type.

The shortest driving distance from the patients' homes to their site of care was calculated in kilometers using ArcView 3.3 by Environmental Systems Research Institute, Inc., and a geographic data set purchased from TeleAtlas, Inc. Driving distance was defined as the shortest distance along roads and highways [8].

### Physical characteristics

Height was measured using a portable stadiometer (SECA, Inc.), weight with a portable scale (LB Dial Scale HAP200KD-41, Healthometer, Inc.), and blood pressure with an automated sphygmomanometer (Omron Model HEM-711). Blood pressure was obtained in the seated position in the left arm (unless contraindicated), using the cuff size recommended by the manufacturer. Three readings were obtained at 5-minute intervals and were averaged for the final result. Body mass index was calculated as weight in kilograms divided by height in meters squared.

### Laboratory results

Glycosolated hemoglobin A1C was measured at 13 clinical laboratories in the patients' home communities. All laboratories used the same high-pressure liquid chromatography method with identical reference ranges. Serum creatinine, urine microalbumin-to-creatinine ratio, total cholesterol, high density lipoprotein cholesterol, and triglycerides were likewise measured by the laboratories. Low density lipoprotein cholesterol (LDL) was calculated using the Friedwald formula (LDL = Total cholesterol - high density lipoprotein cholesterol - triglycerides/5) [9] from fasting specimens. Each patient was classified as being above or below certain laboratory value thresholds recommended by the American Diabetes Association (A1C >8%; A1C <7%; microalbumin-to-creatinine ratio <30 mg/mmol) [10]. If the LDL was 100 mg/dl or greater, or if it could not be calculated because the triglycerides were above 400 mg/dl, we categorized lipids as above goal. Tests were ordered by the primary care provider when clinically indicated. We report the most recent laboratory assays done before the home visit.

### Quality of care

Where possible, we classified each subject as meeting or not meeting recommendations for care made by the American Diabetes Association [10] and the Vermont Program for Quality in Health Care [11]. Creatinine and urine microalbumin tests were due every year. A1C was on time if the latest test was within 3 months (6 months if the latest result was <7.0%). Lipid testing was on time if the latest test was within 6 months (12 months if the latest result showed LDL-cholesterol under 100 mg/dl). Additional measures indicate if the subject was both on time and had results on target for A1C and LDL. Pneumonia vaccine was recommended once ever. Influenza vaccine was considered up to date if the patient reported it was given in the current or previous calendar year.

### Health habits

Alcohol consumption was measured by asking: "How many drinks of the following alcoholic beverages do you have in a typical week (including weekends)?

Bottles or cans of beer: _________

Glasses of wine or wine coolers: _________

Mixed drinks or shots of liquor: _________"

Subjects who indicated that they do not currently drink alcohol were assigned zero to each of the three beverage categories. A summary variable representing total consumption was constructed as the sum of the three beverage-specific responses. Subjects were also asked the four CAGE screening questions [12].

Tobacco use was assessed by asking: "Have you smoked a cigarette – even one puff – during the past seven days?" Those responding "yes" were asked "How many cigarettes do you smoke on an average day?"

### Self care

We assessed self-care behavior with the Summary of Diabetes Self Care Activities Measure [13]. This instrument asks the subject to record how many days in the last week they performed recommended self-care activities such as following a healthful eating plan, or participating in at least 30 minutes of physical activity. Eleven items are used to generate 5 summary scores representing the fraction of days the subject performs recommended activities related to general diet, diabetes-specific diet, exercise, blood glucose self-monitoring, and foot care. Each score ranges from 0 to 100.

### Literacy

The Short Test of Functional Health Literacy in Adults (STOFHLA) is a 7-minute timed instrument that measures the ability to read health-related material [14,15]. The score ranges from 0 to 36 items answered correctly. Responses can be categorized at "inadequate" (STOFHLA 0–16), "marginal" (STOFHLA 17–22), and "adequate" (STOFHLA 23–36).

### Comorbidity

The Self-Administered Comorbidity Questionnaire is a modification of the widely used Charlson Index. It uses patient interview or questionnaire responses rather than chart abstraction for assessment of comorbidity and has excellent agreement with the chart-based Charlson Index [16,17]. We calculated the rate of endorsement of each of 18 specific conditions as well the number of conditions endorsed. We also calculated a score with one point if the condition is endorsed and additional points if the subject reports currently receiving treatment for it, or if it limits activities. Each condition may, therefore, contribute 0 to 3 points for a possible maximum of 54 points. One of the conditions, "eye, nerve, or kidney damage due to diabetes" may be considered a complication of diabetes rather than strictly a comorbidity.

### Functional status and depression

The Medical Outcomes Trust SF-12 Health Survey is a widely used, validated instrument for assessment of general (rather than disease-specific) functional status [18]. Two summary scales are calculated: the Physical Component Summary and the Mental Component Summary. The Patient Health Questionnaire-9 is a brief self-report instrument that quantifies the presence and degree of mental depression [19].

### Complications

We assessed the presence of diabetes complications by asking six questions. The responses were "Yes," "No," and "Don't know."

1. Have you ever had an ulcer or sore on your leg or foot that took more than 4 weeks to heal?

Has your doctor or health care provider ever told you that you have these problems:

2. Problems with vision or retinopathy related to your diabetes?

3. Pain, burning, or numbness in the feet or legs related to your diabetes?

4. Problems with stomach emptying related to your diabetes?

5. Problems with sexual function?"

6. Problems with your kidneys related to your diabetes?

### Medications

The subjects were asked to produce "all medications you have used in the past month including prescriptions, over-the-counter products, vitamins, and herbs." The field assistant recorded the name, strength, dose, route, and frequency of each preparation.

### Quality of life

The Audit of Diabetes-Dependant Quality of Life is an 18-item questionnaire regarding the impact of diabetes on specific aspects of a person's life with patient weighting of the impact of each domain [20,21]. We employed 17 of the 18 domains of this instrument. The scores for each domain can range from -9 (maximum negative impact of diabetes on that domain) to +9 (maximum positive impact).

### Resource utilization

The survey included items asking the subjects to record whether they had used various services in the last year: Endocrinologist, Dietician, Podiatrist, Diabetes Educator, Ophthalmologist, and Diabetes Class. Those answering "Don't Know" were assigned a value of "No." It also prompted subjects to report the number of Emergency Room visits they had in the last year and "In the past month, how many times have you been to a doctor or health care professional?"

### Statistical analyses

In the random effects model, the ICC is the proportion of the total variance that is between clusters (practices).

ICC=σb2/(σb2+σw2)
 MathType@MTEF@5@5@+=feaafiart1ev1aaatCvAUfKttLearuWrP9MDH5MBPbIqV92AaeXatLxBI9gBaebbnrfifHhDYfgasaacH8akY=wiFfYdH8Gipec8Eeeu0xXdbba9frFj0=OqFfea0dXdd9vqai=hGuQ8kuc9pgc9s8qqaq=dirpe0xb9q8qiLsFr0=vr0=vr0dc8meaabaqaciaacaGaaeqabaqabeGadaaakeaacqWGjbqscqWGdbWqcqWGdbWqcqGH9aqpiiGacqWFdpWCdaqhaaWcbaGaemOyaigabaGaeGOmaidaaOGaei4la8IaeiikaGIae83Wdm3aa0baaSqaaiabdkgaIbqaaiabikdaYaaakiabgUcaRiab=n8aZnaaDaaaleaacqWG3bWDaeaacqaIYaGmaaGccqGGPaqkaaa@4137@

where σb2
 MathType@MTEF@5@5@+=feaafiart1ev1aaatCvAUfKttLearuWrP9MDH5MBPbIqV92AaeXatLxBI9gBaebbnrfifHhDYfgasaacH8akY=wiFfYdH8Gipec8Eeeu0xXdbba9frFj0=OqFfea0dXdd9vqai=hGuQ8kuc9pgc9s8qqaq=dirpe0xb9q8qiLsFr0=vr0=vr0dc8meaabaqaciaacaGaaeqabaqabeGadaaakeaaiiGacqWFdpWCdaqhaaWcbaGaemOyaigabaGaeGOmaidaaaaa@30E2@ is the between-cluster component of variance while σw2
 MathType@MTEF@5@5@+=feaafiart1ev1aaatCvAUfKttLearuWrP9MDH5MBPbIqV92AaeXatLxBI9gBaebbnrfifHhDYfgasaacH8akY=wiFfYdH8Gipec8Eeeu0xXdbba9frFj0=OqFfea0dXdd9vqai=hGuQ8kuc9pgc9s8qqaq=dirpe0xb9q8qiLsFr0=vr0=vr0dc8meaabaqaciaacaGaaeqabaqabeGadaaakeaaiiGacqWFdpWCdaqhaaWcbaGaem4DaChabaGaeGOmaidaaaaa@310C@ is the within-cluster component. If a measurement varies across patients without regard to which practice they are in, the ICC will be close to zero. If the value of the variable is largely a function of which practice they are in, the ICC will be close to 1.0 [2]. We used the analysis of variance estimator [22-24] provided by the "loneway" command in STATA 8.2 (Stata Corp., College Station, Texas). This estimator uses the F statistic to calculate the ICC for N total subjects in k groups of size N_o_:

ICC=Fobs−1Fobs−1+g
 MathType@MTEF@5@5@+=feaafiart1ev1aaatCvAUfKttLearuWrP9MDH5MBPbIqV92AaeXatLxBI9gBaebbnrfifHhDYfgasaacH8akY=wiFfYdH8Gipec8Eeeu0xXdbba9frFj0=OqFfea0dXdd9vqai=hGuQ8kuc9pgc9s8qqaq=dirpe0xb9q8qiLsFr0=vr0=vr0dc8meaabaqaciaacaGaaeqabaqabeGadaaakeaacqWGjbqscqWGdbWqcqWGdbWqcqGH9aqpdaWcaaqaaiabdAeagnaaBaaaleaaieGacqWFVbWBcqWFIbGycqWFZbWCaeqaaOGaeyOeI0IaeGymaedabaGaemOray0aaSbaaSqaaiab=9gaVjab=jgaIjab=nhaZbqabaGccqGHsislcqaIXaqmcqGHRaWkcqWGNbWzaaaaaa@41BD@

where

g=N−∑iNo2/Nk−1.
 MathType@MTEF@5@5@+=feaafiart1ev1aaatCvAUfKttLearuWrP9MDH5MBPbIqV92AaeXatLxBI9gBaebbnrfifHhDYfgasaacH8akY=wiFfYdH8Gipec8Eeeu0xXdbba9frFj0=OqFfea0dXdd9vqai=hGuQ8kuc9pgc9s8qqaq=dirpe0xb9q8qiLsFr0=vr0=vr0dc8meaabaqaciaacaGaaeqabaqabeGadaaakeaacqWGNbWzcqGH9aqpdaWcaaqaaiabd6eaojabgkHiTmaaqababaGaemOta40aa0baaSqaaiabd+gaVbqaaiabikdaYaaakiabc+caViabd6eaobWcbaGaemyAaKgabeqdcqGHris5aaGcbaGaem4AaSMaeyOeI0IaeGymaedaaiabc6caUaaa@3E53@

Further,

(V)(ICC)
 MathType@MTEF@5@5@+=feaafiart1ev1aaatCvAUfKttLearuWrP9MDH5MBPbIqV92AaeXatLxBI9gBaebbnrfifHhDYfgasaacH8akY=wiFfYdH8Gipec8Eeeu0xXdbba9frFj0=OqFfea0dXdd9vqai=hGuQ8kuc9pgc9s8qqaq=dirpe0xb9q8qiLsFr0=vr0=vr0dc8meaabaqaciaacaGaaeqabaqabeGadaaakeaadaGcaaqaaiabcIcaOiabdAfawjabcMcaPiabcIcaOiabdMeajjabdoeadjabdoeadjabcMcaPaWcbeaaaaa@3499@

is the asymptomatic standard error of the ICC, and the 100(1-α)% confidence interval is:

ICC±zα/2(V)(ICC).
 MathType@MTEF@5@5@+=feaafiart1ev1aaatCvAUfKttLearuWrP9MDH5MBPbIqV92AaeXatLxBI9gBaebbnrfifHhDYfgasaacH8akY=wiFfYdH8Gipec8Eeeu0xXdbba9frFj0=OqFfea0dXdd9vqai=hGuQ8kuc9pgc9s8qqaq=dirpe0xb9q8qiLsFr0=vr0=vr0dc8meaabaqaciaacaGaaeqabaqabeGadaaakeaacqWGjbqscqWGdbWqcqWGdbWqcqGHXcqScqWG6bGEdaWgaaWcbaacciGae8xSdeMaei4la8IaeGOmaidabeaakmaakaaabaGaeiikaGIaemOvayLaeiykaKIaeiikaGIaemysaKKaem4qamKaem4qamKaeiykaKcaleqaaOGaeiOla4caaa@3FDF@

For each characteristic, we recorded the sample size (N), the sample size per cluster (N_o_) the mean (or proportion for dichotomous variables), the standard deviation (SD) for continuous variables, the standard error of the mean (or percentage) adjusted for clustering within practices (SE), the ICC, and the 95% confidence interval of the ICC. We assessed the association between the value (reported proportion) of binary variables and the ICC [25] with Spearman's non-parametric correlation coefficient. For proportions greater than 0.5, we used the complement of the proportion so that all proportions for this analysis were less than 0.5. To compare groups of ICCs, we used the two-sample Wilcoxon rank-sum (Mann-Whitney) test.

## Results

The results appear in Table 1. The 112 ICCs ranged from 0 for 15 variables with negative values truncated at zero to 0.288 for the proportion with a creatinine measurement on time. The median value was 0.0185 with an inter-quartile range of (0.006, 0.037). Results were similar for 62 binary variables (median 0.022; IQR 0.006, 0.040) and 50 continuous variables (median 0.017; IQR 0.006, 0.032). A Wilcoxon rank-sum test gave a *P *value of 0.54 for the comparison between ICCs of continuous and binary variables.

**Table 1:** Descriptive statistics and intra-practice correlation coefficients

Variable	N	N_o_	Mean or percent	SD	SE	ICC	95% confidence interval
**Demographic, social and economic characteristics**							
Sex (% male)	8808	119.7	48.4		1.3	0.038	0.021, 0.056
Age (years)	8286	117.3	63.1	13.9	0.5	0.077	0.045, 0.109
Married (%)	1004	14.5	62.5		1.6	0.009	0, 0.036
High School Graduate (%)	999	14.4	75.4		1.6	0.011	0, 0.038
Income <$30,000 per year (%)	931	13.4	59.0		2.1	0.042	0.003, 0.081
Private insurance (%)	1001	14.4	58.4		2.0	0.050	0.011, 0.090
Medicare (%)	997	14.4	59.9		1.7	0.018	0, 0.047
Medicaid (%)	995	14.3	21.4		1.8	0.036	0, 0.071
Military or veterans' insurance (%)	994	14.3	5.2		0.8	0.029	0, 0.062
No insurance (%)	993	14.3	2.4		0.6	0.035	0, 0.070
Travel distance from home to Primary Care provider (km)	2955	76.5	13.7	15.3	1.8	0.196	0.102, 0.289
Non-Hispanic white (%)	1004	14.5	97.3		0.5	0.003	0, 0.028

**Physical characteristics**							
Heart Rate (beats per min)	998	14.4	75.0	13.0	0.5	0.015	0, 0.043
Systolic blood pressure (mmHg)	999	14.4	140.3	19.6	0.8	0.042	0.005, 0.079
Diastolic blood pressure (mmHg)	999	14.4	78.3	10.5	0.4	0.017	0, 0.047
Blood pressure below 130/80 mmHg (%)	999	14.4	25.0		1.6	0.028	0, 0.061
Blood pressure over 140/90 mmHg (%)	999	14.4	49.2		2.3	0.069	0.024, 0.114
Height (cm)	998	14.4	165.2	10.4	0.4	0.019	0, 0.048
Weight (kg)	997	14.4	203.0	47.7	1.6	0.011	0, 0.038
Body Mass Index (kg/m^2^)	994	14.3	33.8	7.4	0.2	0.011	0, 0.039
Body Mass Index >30 kg/m^2 ^(%)	994	14.3	67.2		1.6	0.010	0, 0.038

**Laboratory results**							
Mean glycosolated hemoglobin (A1C) (%)	8711	118.4	7.01	1.45	0.05	0.055	0.032, 0.079
A1C >8.0% (%)	8711	118.4	18.1		1.0	0.025	0.013, 0.037
A1C <7.0 (%)	8711	118.4	61.7		1.6	0.046	0.026, 0.066
Total cholesterol (mg/dl)	8167	112.5	184.4	41.8	1.2	0.037	0.020, 0.054
LDL-cholesterol (mg/dl)	7834	111.0	105.8	34.2	1.0	0.045	0.025, 0.065
LDL <100 mg/dl (%) *	7873	111.6	43.8		1.4	0.029	0.020, 0.055
Triglycerides (mg/dl)	7969	109.8	190.5	161.3	3.3	0.014	0.006, 0.022
Serum creatinine (mg/dl)	8474	115.1	1.12	0.70	0.02	0.080	0.048, 0.112
Urine microalbumin to creatinine ratio (mg/mmol)	3039	46.7	47.0	260.7	10.0	0.101	0.051, 0.152
Urine microalbumin to creatinine ratio <30 mg/mmol (%)	3338	49.0	67.3		1.5	0.031	0.011, 0.051

**Quality of care (Process Measures)**							
Creatinine on time (%)	8808	119.7	79.4		3.1	0.288	0.203, 0.373
Urine microalbumin to creatinine ratio on time (%)	8808	119.7	24.6		2.3	0.162	0.105, 0.219
A1C on time (%)	8808	119.7	50.7		2.4	0.118	0.074, 0.162
Lipids on time (%)	8808	119.7	70.3		3.0	0.199	0.132, 0.265
A1C on time & <7% (%)	7308	113.2	35.5		1.5	0.040	0.021, 0.060
LDL on time & <100 mg/dl (%)	7308	113.2	31.3		1.4	0.040	0.021, 0.059
Influenza vaccine given (%)	995	14.3	80.5		1.4	0.058	0.016, 0.100
Pneumonia vaccine given (%)	913	13.2	70.6		1.8	0.058	0.014, 0.102

**Health habits**							
Alcoholic drinks per week	988	14.2	1.6	4.5	0.2	0.027	0, 0.060
CAGE score (0–4)	946	13.6	0.2	0.7	0.2	0.009	0, 0.038
Tobacco smoker (%)	1006	14.5	16.9		1.2	0.005	0, 0.030
Cigarettes per day	159	2.6	17.2	11.2	1.0	0.086	0, 0.270

**Summary of Diabetes Self Care Activities Measure**							
General diet score	953	13.7	58.4	33.3	1.2	0.017	0, 0.048
Specific diet score	980	14.1	52.2	23.9	0.8	0.011	0, 0.038
Exercise score	992	14.3	34.6	32.7	1.2	0.029	0, 0.062
Blood glucose self-monitoring score	933	13.5	57.9	38.9	1.5	0.051	0.010, 0.093
Foot care score	990	14.3	44.2	35.5	1.2	0.002	0, 0.026

**Literacy: Short Test of Functional Health Literacy in Adults**							
Test score (0–36 points)	1002	14.4	29.7	9.6	0.3	0.017	0, 0.079
Inadequate (0–16) (%)	1002	14.4	10.5		1.0	0.011	0, 0.039
Adequate (23–36) (%)	1002	14.4	82.9		1.5	0.037	0.002, 0.073

**Comorbidity**							
Congestive heart failure (%)	1006	14.5	17.1		1.4	0.025	0, 0.057
Coronary artery disease (%)	1006	14.5	19.3		1.4	0.024	0, 0.056
Peripheral vascular disease (%)	1006	14.5	8.7		1.0	0.006	0, 0.032
Stroke (%)	1006	14.5	11.7		1.1	0.006	0, 0.032
Dementia (%) †	1006	14.5	0.9		0.3	0 §	0, 0.024
Asthma (%)	1006	14.5	20.2		1.4	0.014	0, 0.043
Arthritis (%)	1006	14.5	14.1		1.1	<0.001	0, 0.024
Peptic ulcer disease (%)	1006	14.5	14.3		1.2	0.012	0, 0.040
Cirrhosis (%)	1006	14.5	1.9		0.3	0 §	0, 0.024
Paralysis (%)	1006	14.5	3.0		0.6	0.006	0, 0.032
Renal disease (%)	1006	14.5	5.0		0.6	0 §	0, 0.024
Microvascular complications of diabetes (%)	1006	14.5	16.9		1.2	0.012	0, 0.040
Cancer (%)	1006	14.5	11.9		1.0	0 §	0, 0.024
Leukemia (%) †	1006	14.5	0.6		0.2	0 §	0, 0.024
Lymphoma (%) †	1006	14.5	0.5		0.3	0.018	0, 0.048
Metastatic cancer (%) †	1006	14.5	0.7		0.3	0 §	0, 0.024
HIV disease (%) †	1006	14.5	0.1		0.1	0 §	0, 0.024
Depression (%)	1006	14.5	35.0		1.7	0.007	0, 0.033
Comorbid condition count	1006	14.5	1.8	1.7	0.1	0.031	0, 0.065
Self-reported comorbidity questionnaire score (0–54)	1006	14.5	3.6	3.9	0.2	0.032	0, 0.066
Cardiovascular disease (%)	1006	14.5	30.9		1.6	0.018	0, 0.048

**Functional status**							
SF-12 Physical Component Summary score (0–100)	986	14.2	41.2	12.4	0.5	0.028	0, 0.062
SF-12 Mental Component Summary score (0–100)	986	14.2	50.0	10.7	0.4	0.032	0, 0.061
Patient Health Questionnaire-9 depression score (0–27)	591	11.7	4.1	5.0	0.2	0.006	0, 0.044

**Complications of diabetes**							
Foot ulcer (%)	992	14.3	11.3		1.0	0.009	0, 0.036
Vision problems (%)	950	13.7	20.1		1.3	0 §	0, 0.025
Foot or leg pain (%)	943	13.6	30.9		1.8	0.023	0, 0.055
Stomach emptying problems (%)	901	13.0	6.2		0.8	0.003	0, 0.030
Sexual problems (%)	923	13.3	26.4		1.4	0 §	0, 0.026
Kidney problems (%)	490	10.3	9.2		1.4	0.040	0, 0.096

**Medications**							
Insulin (%)	1006	14.5	18.6		1.4	0.014	0, 0.042
Oral hypoglycemic agent (%)	1006	14.5	66.6		1.7	0.022	0, 0.053
Total medication count (0–29)	1006	14.5	8.8	4.5	0.2	0.028	0, 0.061
Prescription medication count (0–24)	1006	14.5	6.7	3.8	0.2	0.055	0.014, 0.095
Non-prescription medication count (0–15)	1006	14.5	2.0	2.4	0.1	0.059	0.017, 0.101

**Audit of Diabetes-Dependant Quality of Life**							
Do physically	985	14.2	-1.9	2.6	0.1	0.001	0, 0.025
Confidence in ability	982	14.2	-2.0	2.7	0.1	0.004	0, 0.030
Motivation	981	14.2	-1.9	2.7	0.1	0 §	0, 0.025
Freedom to eat	985	14.2	-2.8	2.9	0.1	<0.001	0, 0.025
Enjoyment of food	986	14.2	-2.5	2.9	0.1	0 §	0, 0.025
Future	984	14.2	-0.6	3.8	0.1	0 §	0, 0.025
Freedom to drink	983	14.2	-1.5	2.4	0.1	0 §	0, 0.025
Dependence	982	14.2	-0.6	2.8	0.1	0.004	0, 0.029
Working life	981	14.2	-1.5	2.3	0.1	0.014	0, 0.042
Family life	985	14.2	-0.6	2.7	0.1	0.035	0, 0.070
Social life	983	14.2	-0.9	2.0	0.1	0 §	0, 0.025
Sex life	978	14.1	-1.0	2.4	0.1	0.020	0, 0.051
Physical appearance	980	14.1	-1.1	2.3	0.1	0.012	0, 0.041
Holidays/Leisure	983	14.2	-1.5	2.5	0.1	0 §	0, 0.025
Travel	981	14.1	-1.6	2.6	0.1	0.022	0, 0.054
Society reaction	977	14.1	-0.5	1.6	0.1	0.026	0, 0.058
Finances	978	14.1	-1.7	2.7	0.1	0.009	0, 0.037
Average weighted impact	990	14.3	-1.5	2.0	0.1	0.004	0, 0.029

**Resource utilization**							
Endocrinologist in last year (%)	971	14.0	15.9		2.0	0.089	0.037, 0.140
Dietician in last year (%)	984	14.2	34.3		1.8	0.018	0, 0.048
Podiatrist in last year (%)	984	14.2	23.8		1.6	0.022	0, 0.054
Diabetes Educator in last year (%)	970	14.0	23.8		1.8	0.041	0.004, 0.078
Ophthalmologist in last year (%)	944	13.6	60.4		2.1	0.041	0.003, 0.079
Diabetes class in last year (%)	999	14.4	35.0		1.9	0.035	0.002, 0.073
Emergency Room visits in last year (mean)	994	14.3	0.64	1.46	0.05	0.015	0, 0.044
Health professional visits in last month (mean)	991	14.3	1.63	2.39	0.08	0.035	0, 0.070

N = Subjects analyzed; N_o _= Number per cluster; SD = standard deviation of mean; SE = standard error adjusted for clustering; ICC = Intra-cluster (intra-practice) correlation coefficient.

*In 39 cases in which LDL could not be estimated because triglycerides were over 400 mg/dl, LDL was assumed to be over 100 mg/dl.

† Because only a few subjects have this condition, the ICC may not be estimated consistently.

§The method used truncates the estimate of ICC (and its CI) at 0 for some values that may be negative.

The ICCs for the 62 binary variables were significantly associated with their proportions (Spearman's correlation coefficient = 0.53; *P *< 0.0001) to a degree sometimes classified as "Large" [26].

## Discussion

These data provide estimates of intra-cluster correlations for 112 patient characteristics relevant to the analysis of adults with diabetes receiving primary care in Vermont and nearby regions of the United States. They may usefully be applied to the design and sample size estimation of future surveys that are clustered on primary care practices.

In the design of clustered-based studies, the ICC may be used to calculate the design effect which is the degree to which the sample size must be inflated above that of a simple random sample to account for the loss of information inherent in the clustered design. If the average number of subjects sampled per cluster is *m*, the design effect is given by:

Design effect = 1 + (*m*-1)·ICC

If *m *or ICC is large, the total number of individual subjects needed may be substantially greater than suggested by a sample size calculation that is not adjusted for clustering. Alternatively, if both *m *and ICC are small, the design effect may be very close to 1.0 indicating that the clustered design does not inflate the sample size.

In the VDIS, the cost of enrolling subjects for laboratory data collection within a cluster, *once the practice was enrolled*, was relatively low. Therefore, large values of *m *(120.7 subjects per practice on average) were not problematic. However, the cost per patient of obtaining interview data was relatively high. Therefore, we reduced the mean sample size per cluster to 14.5 by random sampling within practice. The design effects experienced in VDIS are not representative of those faced in other designs unless they happen to have the same mean cluster sample size as VDIS (which is extremely unlikely). Therefore, unlike previous publications, we have elected not to report design effects. Study designers should use the ICCs and their own estimates of *m *to understand their own design effects.

Campbell et al [27] suggest that ICCs are higher for process measures than for outcomes. We see evidence for this in that the eight quality of care variables (process measures, see Table 1) have a median ICC of 0.088 (IQR 0.049, 0.181) while the ten laboratory outcomes measures have a median ICC of 0.038 (IQR 0.029, 0.055). This difference is significant by Wilcoxon rank-sum test with *P *= 0.013. We note that the nine physical characteristics of the subjects, presumably under less control of the provider than either laboratory results or even process measures, have a median ICC of 0.017 (IQR 0.011, 0.028) and are significantly different than the laboratory measures (*P *= 0.034).

Within practice correlation was most prominent for process measures associated with quality of care. The likelihood of receiving a creatinine measurement on time had the highest ICC (0.288) with other quality of care measures also among the most highly correlated. This may represent that process measures are heavily influenced by the practice style of the practitioners and any office-based procedures (reminders, registries, flow sheets, etc.) that only some practices employ. In a similar vein, physiologic control of some aspects of diabetes (especially achievement of tight control of A1C and LDL) appears to vary importantly across practices with ICCs of 0.046 for A1C below 7% and 0.029 for LDL below 100 mg/dl.

Some demographic aspects of the population were correlated within practices: age, sex, and especially travel burden. As patients tend to stay with their primary provider as they age, some practices accumulate older patients (ICC = 0.077). Some patients express a preference for same-sex providers, with more women visiting practices that have female providers. This may account for the relatively high ICC for sex (0.038). Travel distance may be related to the geographic location of the practice office. Practices in more densely populated areas may tend to have lower typical travel burdens.

With the possible exception of blood pressure (which may be under the control of the providers to some degree), the physical characteristics and health habits of patients vary little across practices.

Although apparently under the control of the providers, the utilization of health care services had generally small ICCs. The exception was consultation by an Endocrinologist with an ICC of 0.089. This may reflect the geographic proximity of an endocrinologist to some of the practices.

The aspects of diabetes that are most directly experienced by patients (complications, quality of life, functional status, comorbidity, and self-care) vary little across practices. It does not appear that some primary care practices tend to accumulate more complicated or difficult patients than others. Likewise, low health literacy is a substantial, and perhaps unrecognized, problem for all practices, with little clustering within practices.

These data demonstrate a large correlation between the proportion of the 62 binary variables and their ICCs. This finding has been noted by others [25] and may be useful in estimating an ICC for sample size calculations.

For many of these variables, the impact of within-cluster correlation on sample size requirements appears to be relatively small. Thirty-three variables had an ICC <0.010 with a design effect less than 1.14 indicating that the VDIS clustered design required an increase in sample size of 14% compared to a non-clustered study. However, depending on the number of clusters and the number of subjects per cluster, even small ICCs can result in the need for costly increases in overall sample size.

These estimates of ICC are not useful for studies that cluster on factors other than practice (such as community, hospital, individual provider, family, classroom, *etc*.). The VDIS study population was drawn from Vermont and nearby New York and New Hampshire and is, therefore, predominantly white and rural. All the subjects were under care for diabetes. Although some have Type 1 diabetes, this older, overweight population is largely comprised of patients with Type 2 diabetes. The practices in the VDIS are small with a median of 2 providers per practice. For these reasons, generalization to other populations and settings may be problematic.

Several recent reports provide some comparisons from other settings (Table 2). A study of British patients aged 75 and older reported intra-practice correlations from 106 general practices [4]. A study from Rhode Island and nearby Massachusetts enrolled 15 primary care practices [5]. Several surveys of general practices from Australia and New Zealand provided a few ICCs comparable to VDIS [3,6]. The ICCs for most of these variables vary substantially across the studies. For instance, the ICCs for weight and body mass index varied between 0.011 and 0.081. Differences in the practice structures, referral patterns, social and geographic factors and practice patterns may explain these differences. Only recently have determinants of ICC begun to be studied [26-28]. We suggest that more catalogues of ICCs, drawn from a variety of settings, will be useful both to investigators designing new clustered studies and to researchers investigating the role of setting on patient characteristics.

**Table 2:** Intra-practice correlation coefficients from four recent studies

Characteristic	VDIS	BEACH	CEART	MRC	HPOP	JOGS
Age	0.077	0.153–0.159				0.050
Sex	0.038	0.055–0.066				
Height	0.019		0.053	0.048		
Weight	0.011		0.020	0.012		0.043
Body mass index	0.011		0.031	0.022		0.081
Systolic blood pressure	0.042		0.048			0.018
Diastolic blood pressure	0.017		0.129			0.046
Heart rate	0.015			0.032		
Total cholesterol	0.037		<0.001			0.004
LDL-cholesterol	0.045		0.006			
LDL-cholesterol at goal	0.038		0.027			
Triglycerides	0.014		0.024			
Serum creatinine	0.080		0.00007			
Tobacco use	0.005		0.036	0.006		0.055
Number of medications	0.028				0	
Influenza vaccination	0.058				0.062	
Depressed	0.007				0	

VDIS = Vermont Diabetes Information System (the present study) [7].

BEACH = Bettering the Evaluation and Care of Health [3].

CEART = Cholesterol Education and Research Trial [5].

MRC = Medical Research Council Trial of the Assessment and Management of Older People in the Community [4].

HPOP = Health Promotion for Older People [6].

JOGS = Justification of Green Scripts trial [6]

## Conclusion

Intra-practice correlation coefficients in this survey of adults receiving care for diabetes varied widely. Some characteristics (such as the likelihood of having a recent creatinine measurement) were highly associated with the practice (ICC = 0.288), while others (prevalence of some comorbidities and complications and certain aspects of quality of life) varied much more across patients with virtually no correlation within practices (ICC<0.001). The values reported here may be useful in designing future clustered studies.

## Competing interests

The author(s) declare that they have no competing interests.

## Authors' contributions

BL conceptualized the research and performed the analyses. CDM oversaw the data collection and management. Both authors read and approved the final manuscript.

## Pre-publication history

The pre-publication history for this paper can be accessed here:


